# Iron Treatment May Be Difficult in Inflammatory Diseases: Inflammatory Bowel Disease as a Paradigm

**DOI:** 10.3390/nu10121959

**Published:** 2018-12-11

**Authors:** Carla J. Gargallo-Puyuelo, Erika Alfambra, Jose Antonio García-Erce, Fernando Gomollon

**Affiliations:** 1 Digestive Diseases Service, Hospital Clínico Universitario “Lozano Blesa”, Zaragoza, 50009 Aragón, Spain; ealfambra.due@gmail.com (E.A.); fgomollon@gmail.com (F.G.); 2 IIS Aragón, Zaragoza, 50009 Aragón, Spain; 3 Blood and Tissue Bank of Navarra, Pamplona, 31008 Navarra, España; jagarciaerce@gmail.com; 4 School of Medicine, University of Zaragoza, Zaragoza, 50009 Aragón, Spain; 5 Centro de Investigación Biomédica en Red Enfermedades Hepáticas y Digestivas, CIBEREH, 28029 Madrid, Spain

**Keywords:** inflammatory bowel disease, iron deficiency, iron deficiency anemia, intravenous iron, oral iron

## Abstract

Iron plays a key role in many physiological processes; cells need a very exact quantity of iron. In patients with inflammatory bowel disease, anaemia is a unique example of multifactorial origins, frequently being the result of a combination of iron deficiency and anaemia of chronic disease. The main cause of iron deficiency is the activity of the disease. Therefore, the first aim should be to reach complete clinical remission. The iron supplementation route should be determined according to symptoms, severity of anaemia and taking into account comorbidities and individual risks. Oral iron can only be used in patients with mild anaemia, whose disease is inactive and who have not been previously intolerant to oral iron. Intravenous iron should be the first line treatment in patients with moderate-severe anaemia, in patients with active disease, in patients with poor tolerance to oral iron and when erythropoietin agents or a fast response is needed. Erythropoietin is used in a few patients with anaemia to overcome functional iron deficiency, and blood transfusion is being restricted to refractory cases or acute life-threatening situations.

## 1. Introduction

Iron is a key element in life. Organism and cells need a very exact quantity of iron: too much can be toxic and too little is bad for metabolism. Iron deficiency refers to the reduction of iron stores and may progress to iron deficiency anaemia (IDA), which is a more severe condition in which low levels of iron are associated with anaemia. It is important to note than iron deficiency and anaemia are not synonymous, a normal haemoglobin level does not exclude iron deficiency. Individuals with normal body iron stores must lose a large portion of body iron before the haemoglobin falls below the laboratory definition of anaemia. Both IDA and iron deficiency have clinical relevance [1].

Anaemia is both a significant and common clinical problem in inflammatory bowel disease (IBD). If we consider it as an “extraintestinal manifestation” of IBD, anaemia is probably the most frequent, and at least one of the most relevant [2,3,4]. The presence of anaemia independently affects not only the quality of life or the ability to work, but also can be associated with an increased risk of complications or even death [5,6,7]. As a matter of fact, severe anaemia has been linked to worse results after intestinal surgery [8,9]. However, anaemia in IBD does not receive enough attention, since it is both under-diagnosed and under-treated in adults and children; it may be so frequent that sometimes it is considered an inevitable manifestation of IBD. In these patients, anaemia is a unique example of the multifactorial origin: malabsorption of iron, folic acid and/or vitamin B12, blood loss through intestinal lesions, and anaemia caused by inflammation, also called anaemia of chronic disease or anaemia of chronic inflammation (ACD). ACD can be defined as a multifactorial anaemia associated with increased cytokine production, up-regulation of hepcidin, and abnormal iron homeostasis [1]. Most IBD patients with anaemia suffer from an anaemia to which several factors contribute, with the most common being iron deficiency and inflammation [3,4]. This leads to a diagnostic challenge (for example, it may be more difficult to determine the iron deficiency if there is a concomitant ACD) and therapeutic challenge; it is not enough to control the inflammation of the IBD if the iron supplementation is not carried out for effective anaemia treatment.

## 2. Iron Homeostatic Hormone Hepcidin and Its Inflammatory Regulation

Iron plays an important role in many physiological processes. The absence of a defined pathway to excrete excess iron makes it essential for the body to regulate the amount of iron absorbed. This regulation is mediated by the iron-regulatory hormone hepcidin [10]. The delivery of iron to every cell in the body depends on circulating iron. Iron is supplied to the circulation from macrophages recycling senescent erythrocytes, from duodenal enterocytes absorbing dietary iron, and from hepatic stores. All of these cells release iron into the circulation through the only known iron exporter, ferroportin. Therefore, the rate of iron entry into the circulation is proportional to the amount of ferroportin on iron-exporting cells. Hepcidin inhibits the release of iron into the circulation by postranslationally regulating ferroportin, which is expressed on the membrane of duodenal enterocytes, on macrophages, placental syncytiotrophoblasts, and hepatocytes [11,12].

The main factors that are implicated in hepcidin regulation include liver iron stores, blood plasma iron, hypoxia, inflammation and erythropoiesis. Hepcidin synthesis is induced by iron overload and inflammation, and inhibited by anaemia and hypoxia. In inflammatory conditions, circulating proinflammatory cytokines (especially interleukin-6) induce hepcidin production and release. This results in increased internalization and degradation of ferroportin and subsequently sequesters iron in splenic and hepatic macrophages and duodenal enterocytes, preventing export into the plasma. This is thought to be the main pathogenetic mechanism of ACD, and may result in insufficient iron availability to meet the body’s needs [10,11,12,13]. Also, there may be hepcidin-independent mechanisms that may also contribute to hypoferremia of inflammation via the direct suppressive effect of inflammation on ferroportin [14]. As a result, patients with chronic inflammatory conditions have greater daily iron requirements to increase the levels of circulating iron compared with healthy individuals. 

## 3. Prevalence of Anaemia in IBD

Anaemia is common in chronic inflammatory diseases, including IBD, as we mentioned previously [2,3,4,15]. The reported prevalence of anaemia in IBD patients varies noticeably, depending both on the definition and on the specific patient population (hospitalized vs. ambulatory patients, in remission vs. active IBD). Several factors influence haemoglobin levels, such as age, gender, ethnicity, high altitudes, pregnancy and smoking. The currently used WHO definition of anaemia applies also to patients with IBD. Minimum haemoglobin levels used to define anaemia in people living at sea level are: <12.0 g/dL in non-pregnant women, <11.0 g/dL in pregnant women <13.0 g/dL in men [16]. Prevalence in IBD patients has been estimated at between 6 and 74% and, in hospitalized patients, prevalence rates above 50% are repeatedly published [17]. The most frequent cause of anaemia in patients with IBD is iron deficiency, and is present in 13–90% of cases [18], which underlines the fact that this condition may be considered more a rule than an exception in IBD patients. However, the cause of anaemia in many, if not most, IBD patients is chronic inflammation in combination with iron deficiency.

## 4. Iron Deficiency in IBD

An excess of iron can be toxic for the intestinal mucosa and higher iron content in water has been linked in one epidemiological study to the incidence of IBD [19,20,21]. However, the most frequent clinical situation in IBD is iron deficiency, as a consequence of reduced iron intake, impaired iron uptake through the duodenum-jejunal mucosa by inflammation or intestinal resection, and/or continuous blood loss from the ulcerated mucosa [22]. The inflammatory cytokines, IL-1, IL-6, and oncostatin M are known to disrupt intestinal iron absorption via hepcidin-induced ferroportin degradation, independent of the underlying type and location of IBD [10,11,12,13,14]. Moreover, several studies have demonstrated tumour necrosis factor to inhibit duodenal iron absorption via a hepcidin-independent mechanism involving tumour necrosis factor-induced iron storage within ferritin in enterocytes [23,24,25].

Iron deficiency has clinical relevance [26]. A lot of evidence shows that normal iron status is necessary for growth and cognitive development, and quality of life, for example. Patients with iron deficiency without anaemia may have symptoms such as reduced exercise tolerance (iron is required for optimal mitochondrial function essential for respiration and energy production) or fatigue [27,28,29,30,31,32]. In patients with IBD, iron deficiency may induce secondary thrombocytosis which has been identified as an independent risk factor for thromboembolic events [33,34]. In experimental studies, iron deficiency alters megakaryopoiesis and platelet phenotype independently of thrombopoietin, through a mechanism mediated by increased expression of hypoxia-induced factor 2α [35,36]. The correction of iron deficiency lowers platelet counts and platelet activation in IBD-associated secondary thrombocytosis, and might contribute to reduce the increased risk of thromboembolic events in IBD patients [37]. Finally, observational studies have showed that pre-surgery iron deficiency is a predictive factor of poor outcomes in abdominal surgery (increased rates of blood transfusion, post-operative infections and fatigue) [8,9].

## 5. Diagnosis of Anaemia and Iron Deficiency in IBD

The initial workup of anaemia should follow a simple algorithm widely used. Starting from the evaluation of mean corpuscular volume (MCV), microcytosis indicates iron-restricted anaemia (true or functional iron deficiency). MVC is difficult to interpret in azatioprine or mercaptopurine-treated patients, because both medicines increase it [38]. Also, MCV is difficult to interpret in patients with β thalassemia trait, in whom MCV is usually very low, regardless of the existence or not of iron deficiency. The Mentzer index (mean corpuscular volume/red blood cell count) uses the complete blood count to differentiate thalassemia from IDA. A Mentzer index <13 suggests thalassemia, and an index >13 suggests iron deficiency. If a thalassemia is suspected, haemoglobin electrophoresis should be done [39]. Moreover, sometimes in IBD, microcytosis and macrocytosis (due to drugs, B12 or folic acid deficiency) co-exist, so that the two abnormalities may neutralize each other and result in a normal MCV, although iron deficit exists [38]. After MCV, reticulocyte count should be evaluated. Low or ‘normal’ counts show incapability to respond correctly to anaemia, either due to deficiencies that result in inappropriate erythropoiesis or primary bone marrow disease. High reticulocytes count shows increased erythrocytes formation and hence exclude deficiencies. A wide size range of the erythrocytes (high RDW) can help in this situation, as high RDW is an indicator of iron deficiency. Cytometry of reticulocyte haemoglobin content and percentage of hypochromic red cells have shown high predictive value in differential diagnosis of IDA, independent of inflammation and ACD. While a decrease in percentage of hypochromic erythrocytes indicates insufficient long-term iron supply, diminished reticulocyte haemoglobin content signifies current deficit, reflecting iron bioavailability over the previous 3–4 days. Reticulocyte haemoglobin content is also a proven early marker for treatment response to iron supplementation [40,41]. 

**Figure 1:**
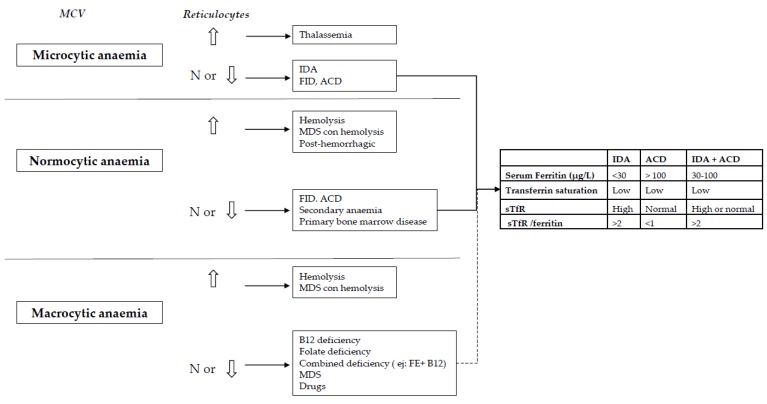
Diagnosis of types of anaemia. MCV: mean corpuscular volume, N: normal, IDA: iron deficiency anaemia, FID: functional iron deficiency, ACD: anaemia of chronic disease, MDS: myelodysplastic syndrome, sTfR: soluble transferrin receptor.

In relation to iron metabolism indices, low iron, low ferritin, low transferrin saturation and high transferrin concentrations indicate iron deficit. However, diagnosing iron deficit in IBD patients may be difficult, especially when both iron deficiency and ACD are present (as previously mentioned, both frequently coexist). In this setting, many of the laboratory measures of iron status may be unreliable as inflammation modifies the parameters of iron metabolism [42,43]. For instance, if there is chronic inflammation, the elevation in transferrin levels typical of iron deficit may not be found as patients with low albumin also tend to have low transferrin concentrations. Moreover, serum ferritin, the most accessible measure of iron stores and the most powerful test for iron deficiency, can be normal or even increase in the presence of iron deficiency, in response to inflammation as it is an acute phase reactant. Therefore, ferritin may not provide adequate information about iron stores in chronic inflammatory diseases such as IBD. Increased soluble transferrin receptor concentration distinguishes reliably between iron deficiency and ACD [44,45,46,47,48,49]. It is elevated when the bone marrow needs more iron, both in elevated erythropoietic activity and in iron deficiency (true or functional). Therefore, a high soluble transferrin receptor (sTfR) is a good indicator of iron-deficient erythropoiesis, especially useful in the identification of iron deficit in patients with inflammation (with normal or even elevated serum ferritin). Also, sTfR-ferritin (TfR/F) ratio may be a useful clinical marker for anaemia when combined with other haematological parameters such as reticulocyte haemoglobin content [44,45,46,47] Figure 1.

Accordingly, the “European Consensus on the Diagnosis and Management of Iron Deficiency and Anaemia in IBD” recommends that the diagnostic criteria for iron deficiency need to be adapted to the level of inflammation [38]. Thus, in patients without biochemical, endoscopic or clinical evidence of active IBD, serum ferritin <30 μg/L is low and defines iron deficiency, but, in patients with active disease, the lower cut-off of serum ferritin consistent with normal iron deposits should be increased up to 100 μg/L. On the other hand, if there is biochemical or clinical evidence of inflammation, the diagnostic criteria for ACD are a serum ferritin >100 μg/L and a soluble transferring receptor <20%. If ferritin is between 30 and 100 μg/L, a combination of true iron deficiency and ACD is presumable [38].

## 6. Treatment

### 6.1. Is it Necessary to Achieve IBD Remission for the Treatment of IDA?

Yes. Our first aim should be to reach IBD clinical remission because the principal causes of iron deficiency in IBD patients are iron malabsorption and blood loss through active intestinal lesions. Although seemingly self-evident, sometimes this step is forgotten in real clinical practice: treatment with iron will be little effective if there is active inflammation and in fact recurrence of anaemia will be frequent, even in those patients with totally filled iron stores and treating with intravenous iron as maintenance [38,50]. However, in many cases, achieving clinical remission is not enough, and iron stores should be filled specifically.

### 6.2. When to Start Iron Supplementation in IBD Anaemic Patients and What Is the Goal?

It should not be assumed that some level of anaemia is a normal finding in IBD patients and consequently need not be treated [4,51,52]. On the contrary, iron treatment should be started as soon as anaemia is detected. In fact, most patients without anaemia but with iron deficiency should be treated to avoid the development of IDA and because, even without anaemia, iron deficiency can have clinical relevance (fatigue, reduced exercise tolerance, etc.). There is solid evidence about the benefit of oral or intravenous iron replacement in chronic fatigue, heart failure, athletes and blood donors [30,38,53,54,55]. 

The aim of iron supplementation is to normalize haemoglobin levels and iron stores. Short-term recurrence is less probable if ferritin levels reached are >100 μg/L [56].

### 6.3. What Is the Best Method of Iron Supplementation for IBD Patients? Oral or Intravenous Route?

The supplementation route should be determined according to symptoms, severity of iron deficiency and/or anaemia and taking account of comorbidities and individual risks associated with therapy.

#### 6.3.1. Oral Iron in IBD

Iron deficiency has been treated with different formulations of oral iron for centuries, and in fact clinical observational and controlled studies prove that oral treatment can be effective in IBD. However, it is far from ideal in IBD patients, and has relevant limitations:

(1) The absorption of oral iron can be severely compromised because of the gut inflammation and, in some Crohn’s patients, because of prior intestinal resections or duodenal involvement [57,58].
(2) Oral iron is often poorly tolerated by patients. The intolerance rate (mainly nausea, abdominal pain, or diarrhoea) is a frequent finding leading to discontinuation in up to 50% of patients [59,60]. Moreover, it is important to note that most IBD patients are receiving several drugs, and these side effects of oral iron could negatively affect the overall compliance [61]. In point of fact, it is plausible that bias partially explains oral iron efficacy in clinical trials, since patients with poor oral iron tolerance are probably under-represented in this setting.
(3) Oral iron may be slow in filling iron stores and recovering anaemia. It can require months. A more rapid response is advisable in severe-moderate cases, particularly to make easy the fast return of the patient to an active way of life. Furthermore, in some patients, persistent intestinal blood loss is greater than the intestinal absorption of iron [18].
(4) Finally, despite its crucial role in cellular processes, free colonic iron can generate toxic free radicals and reactive oxygen species, which can directly affect gut epithelial integrity via the promotion of redox stress [62]. Experimental evidence suggests that the excess of luminal iron can be harmful for the gut mucosa. In vitro studies with Caco-2 cells exposed to iron have shown an impaired epithelial integrity [63,64]. In several animal studies, free luminal iron has been shown to be directly harmful, pro-inflammatory, and even to favour carcinogenesis [19,65,66]. Wermer et al. show that free luminal iron favoured inflammation in the terminal ileum due to disturbances in the intestinal microbiota in a murine experimental model of IBD. On the contrary, intravenous iron did not result in intestinal mucosa lesions in the murine experimental model [67]. There is only one open-label clinical trial that has compared the effects of oral and intravenous iron replacement therapy on the gut microbiome and metabolome in patients with IBD. In this study, both oral and intravenous iron improved iron deficiency, but higher serum ferritin levels were reached with intravenous iron. Noticeable shifts in gut bacterial diversity were described in patients with IBD and iron deficiency following oral iron supplementation (however, other changes were also observed following intravenous treatment) and the gut microbiome in turn might have an effect on disease activity in IBD [68].

Therefore, although oral iron is the mainstay therapy for iron deficiency in several scenarios, in IBD patients, oral iron supplements only can be used in patients with mild anaemia, defined by the WHO as haemoglobin 11.0–11.9 g/dL in non-pregnant women and 11.0–12.9 g/L in men, whose disease is inactive and who have not been previously intolerant to oral iron [38]. There is a big variety of formulations of ferrous salts (sulphate, gluconate, fumarate, etc.) and ferric complexes (amino acids, polysaccharide, ovo-albumin, etc.). There is no evidence that one of them is more effective or has fewer side effects than another, as long as they are taken. Only and because they are poorly absorbed, the use of enteric-coated formulations should be avoided [69]. Although conventional wisdom “says” that up to 200 mg of elemental iron per day is required to correct IDA [70], this is probably incorrect. A maximum of 10–20 mg of oral iron can be absorbed per day in the gut. In this respect, a single tablet of most of the ferrous salts formulations provides more iron than the gut is able to absorb per day. Non-absorbed iron salts can be harmful, and in any case high doses of oral iron may cause more side events [66,71,72,73,74,75,76]. Moreover, from a clinical point of view, it is not possible to differentiate the typical side effects of oral iron (nauseas, diarrhoea, constipation, etc.) from IBD symptoms, which is a real problem in clinical practice. Finally, no absorbed iron salts may inhibit (e.g., feedback) the intestinal iron absorption and decrease the tolerance. In an interesting randomised study, 90 patients with IDA (average age 85 years) received 15 mg, 50 mg or 150 mg of elemental iron/day. At two months, there were no between-group differences in the levels of haemoglobin or ferritin, but adverse side effects were significantly more frequent with higher doses [77]. There was also an observational study of patients with IBD with mild-to-moderate anaemia that shows that oral iron treatment at low doses (100 mg/day) was effective and well tolerated by most patients, did not exacerbate the symptoms of the underlying IBD, and was associated with a relative improvement in quality of life [78]. Moretti et al. in their recent study confirmed that in women with iron deficiency, iron absorption is highest at lower iron doses (40–80 mg), and also that low doses of iron given on alternate days may maximise fractional iron absorption, increase dosage efficacy and decrease gastrointestinal adverse effects [79]. Their results show the need to study longer-term, alternate-day schedules for oral iron treatment in IBD patients. In summary, as absorption and efficacy of oral iron are no greater with high iron doses, and because adverse effects are dose-related, oral iron should be recommended in low doses (e.g., 50–100 mg of elemental iron daily) in IBD patients. After normalisation of haemoglobin levels, oral iron treatment must persist for at least 3 months to completely replenish iron stores [80]. In clinical practice, non-responders to oral iron should be switched to intravenous iron. Although there is scarce evidence, probably a haemoglobin increase of ≥1.0 g/dL at day 14 after commencement of oral iron replacement may be the most accurate predictor of sustained treatment response, with a positive predictive value of 92.9% [81].

#### 6.3.2. Intravenous Iron in IBD

The use of intravenous iron is not new, its efficacy as a treatment of iron deficiency or IDA was demonstrated in inflammatory chronic diseases in the first half of the twentieth century, especially in rheumatoid arthritis [82]. However, with the preparations available in that moment of high molecular weight iron dextran, severe toxicity (including death) was rather common and unpredictable, and because of that, its use was never very popular [83]. However, since the end of the twentieth century, several studies that included thousands of patients have showed the efficacy and safety of new preparations of intravenous iron in very different clinical settings. With new products, which release less elemental iron into the bloodstream, serious anaphylactic-type reactions are very rare [82,84]. Chertow et al. found absolute rates of life-threatening adverse reactions of 0.6, 0.9, 3.3 and 11.3 per million infusions for iron sucrose (IS), sodium ferric gluconate complex (FG), low molecular weight (LMWID), and high molecular weight iron dextran, respectively [85]. Recently, Wang et al. compared the risks of anaphylaxis associated with intravenous iron dextran, gluconate, sucrose, or ferumoxytol (FXT) and found IS to have the lowest and iron dextran the highest risk [86]. In another study, while high molecular weight iron dextran and ferric carboxymaltose (FCM) were found to be similarly effective, FCM was associated with fewer hypersensitivity reactions [87]. The ligands of FXT and iron isomaltoside 1000 (ISM) have been shown to cross-react with antidextran antibodies in vitro. Therefore, both should be used with caution in patients who have previously shown intolerance to iron dextran [88].

Studies in IBD patients have not reported specific side effects of intravenous iron in this clinical scenario. Although minor side effects are frequently shown in clinical trials, serious adverse events are very scarce; for example, in some retrospective studies including millions of treatments, IS was linked with less than one serious adverse event in 100,000 treatments [89]. The superior efficacy of intravenous iron over oral iron for the treatment of IDA and maintenance of iron stores in IBD has already been shown in four separate meta-analyses [90,91,92,93]. Intravenous administration has been found to achieve higher ferritin levels than oral iron, thus possibly reducing the likelihood of anaemia recurrence in the long term. Moreover, intravenous iron shows a faster response, is better tolerated than oral iron, and is safe. Thus, intravenous iron is recommended by the “European Consensus on the Diagnosis and Management of Iron Deficiency and Anaemia in IBD” as a first line treatment in patients with active IBD, with prior poor oral iron tolerance, with haemoglobin <10 g/dL, and in patients who need erythropoiesis-stimulating agents [38]. 

Six intravenous iron formulations are currently available for treatment of IDA worldwide: ferric gluconate (FG), iron sucrose (IS), low molecular weight iron dextran (LMWID), ferric carboxymaltose (FCM), iron isomaltoside-1000 (ISM), and ferumoxytol (FXT). In an overall setting, FCM is the intravenous iron formulation for which there is the highest level of clinical evidence. In IBD, large published trials are available from IS, FCM and ISM. Recommendations on dosage and infusion rate for the different formulations vary considerably. For FCM, the maximum dose is 1000 mg (or 20 mg/kg)/once weekly infused over 15 min; for IS and LMWID, 100–200 mg, maximum 3 times per week, infused over 1 h; and for ISM, 20 mg/kg, maximum also 3 times per week, infused over 15–30 min, [89]. We want to highlight:

- There is little experience with LMWID; moreover, a test dose is necessary, and in most cases it requires several hours to infuse the total dose, compared with shorter times for other preparations. In consequence, at the moment, this preparation cannot be recommended in IBD patients.
- IS has a dose limitation: a maximum dose of 200 mg per infusion can be given to prevent the release of potentially toxic free iron. As the iron deficit in IBD is frequently in the 1000–2500 mg range, usually 3 to 10 one-hour infusions are needed. By contrast, a dose of 1000 mg of FCM can be given in a single 15-min infusion. In 2011, Evstatiev and colleagues published a randomized, controlled non-inferiority trial to compare the use of FCM versus IS in out-patients with IBD and anaemia [94]. Both formulations were effective, well-tolerated, and demonstrated similar improvements in quality of life and in physical and mental components or more-specific IBD-related indexes. However, FCM was markedly better in correcting anaemia than IS; more patients on FCM had their haemoglobin levels increase by ≥20 g/L or achieved normalization than with IS. Moreover, FCM was much more suitable for patients. Of course, on a weight-by-weight basis, FCM formulation is more expensive than IS, but from an entire cycle of care perspective, FCM was cheaper.
- FXT is not approved in Europe for treatment of IDA in IBD patients, because it can interfere in the realization of magnetic resonance, a very useful imaging technique in our patients [38].

A recent systematic review with network meta-analysis published in 2017 compared the efficacy and tolerability of different intravenous iron formulations in IBD patients. All intravenous formulations were more effective than oral iron, but this difference was statistically significant only for FCM, and a rank probability matrix indicated FCM to be the most effective intravenous iron formulation, followed by ISM and IS. In relation to safety, adverse events rates were 12%, 15%, 12% and 17% for FCM, IS, LMWID and ISM, respectively. One drug-related severe side-event each was reported for FCM and ISM, and one possible drug-related serious adverse event for IS. Moreover, because of the possibility of a quick and high-dose infusion, FCM is similar to IS from an economic point of view. Higher costs of FCM are compensated by savings in practice and clinic overhead costs, patient travel and costs for time lost from work [93].

Finally, we want to emphasize that premedication with antihistamines does not prevent the rare infusion reactions produced by current intravenous iron formulations, and should be proscribed. The European Medicines Agency recommends only close monitoring for signs of hypersensitivity during and for at least 30 min after every administration of an intravenous iron formulation, especially for patients at risk, such as those who have had a previous adverse reaction to intravenous iron or have more than one drug allergy, a history of severe atopy, pre-existing severe respiratory or cardiac disease, or are taking beta-blockers or angiotensin-converting enzyme inhibitors. High-molecular weight iron dextran, which produced hypersensitivity reactions more frequently than any other intravenous iron formulation, is no longer available [95].

### 6.4. How Do We Estimate the Necessary Amount of Iron?

In past general practice, iron needs were habitually estimated using the Ganzoni formula [iron deficit = body weight x (target Hemoglobin–current Hemoglobin) × 2.4 + iron store (500 mg for body weight greater than or equal to 35 kg and 15 mg/kg for body weight less than 35 kg)]. However, several experts asserted that iron needs were undervalued, and that response could get better with higher cumulative doses. A simpler fixed-dose regimen based on haemoglobin and body weight applied to FCM dosing in IBD patients showed superior efficacy compared with IS dosing according to the Ganzoni formula [38,94]; see Table 1. This simple dosing table now provides the basis for dosing recommendations for both FCM and ISM. For patients requiring fast and efficient iron replenishment, high-dosed FCM and ISM are generally favoured, since these formulations have been more comprehensively tested at high doses in clinical and observational trials.

Even after anaemia correction and iron store repletion, anaemia recurs in over 50% of IBD patients within 10–12 months [94]. This recurrence can effectively be prevented [96,97,98]. In IBD patients, haemoglobin and iron status markers should be monitored every 3 months for at least one year after correction, and every 6–12 months after normalisation of haemoglobin. After successful treatment of IDA with intravenous iron, re-treatment with intravenous iron should be got started as soon as serum ferritin falls below 100 μg/L or haemoglobin below 12 or 13 g/dL (according to gender).

### 6.5. When to Use Erythropoiesis-Stimulating Agents and/or Blood Transfusion?

Intestinal inflammation is mediated by cytokines, which may contribute to the generation of anaemia in chronic disease, accompanied by inadequate erythropoietin production. Thus, IBD-associated anaemia is a unique example of the combination of chronic iron deficiency and anaemia of chronic diseases. ‘Functional’ iron deficiency—defined as an inappropriate availability of iron for erythropoiesis despite normal body iron stores—is a direct consequence of this complex pathogenesis in many patients. In the majority of patients with IBD, treatment of the underlying inflammatory condition, together with adequate iron treatment, is sufficient to correct IDA [99]. Patients with ACD with an insufficient response to intravenous iron and despite optimized IBD treatment may be considered for Erythropoiesis-stimulating agents with a target haemoglobin not above 12 g/dL. Erythropoietic agents should always be combined with intravenous iron treatment, because functional iron deficiency is likely to appear [100]. In patients with Crohn’s disease, both folic acid and vitamin B12 should also be regularly evaluated, and if there are deficiencies, they should be treated.

Red blood cell transfusion should generally be considered only when haemoglobin concentration is 7 g/dL, in the presence of severe comorbidities or other individual risk factors, or when facing a life-threatening situation. Blood transfusions should be followed by subsequent intravenous iron supplementation [38].

## 7. Conclusions

Iron deficiency without anaemia and IDA are both common in IBD, and accurate diagnosis and treatment of them is essential. In the treatment of IDA in patients with IBD, it is very important to both control intestinal inflammation and correct the iron deficiency. Oral iron supplementation can be used in patients with mild anaemia and without inflammatory activity, but intravenous iron should be chosen as the first line of treatment in patients with haemoglobin < 10 g/dL, in patients with active IBD, in patients with low tolerance to oral formulations and when erythropoietin agents or a fast response is needed. Intravenous iron formulations can be chosen depending on local availability and patient convenience. Erythropoietin is needed (combined with intravenous iron) in a few patients with anaemia to get over functional iron deficiency, and blood transfusion is being restricted to refractory cases or acute life-threatening situations.

## Figures and Tables

**Table 1:** Simple estimation of total iron need in patients with anaemia (Hb (g/dL) <13 in men, <12 in women).

	Body Weight ≥70 kg	Body Weight <70 kg
Anaemia with Hb > 10 g/dL	1500 mg	1000 mg
Anaemia with Hb 7–10 g/dL	2000 mg	15000 mg
Anaemia with Hb < 7 g/dL	Probably 2500 mg	Probably 2000 mg
